# Role of p53 in the progression of gastric cancer

**DOI:** 10.18632/oncotarget.2434

**Published:** 2014-09-03

**Authors:** Rita A. Busuttil, Giada V. Zapparoli, Sue Haupt, Christina Fennell, Stephen Q. Wong, Jia-Min B. Pang, Elena A. Takeno, Catherine Mitchell, Natasha Di Costanzo, Stephen Fox, Ygal Haupt, Alexander Dobrovic, Alex Boussioutas

**Affiliations:** ^1^ Cancer Genetics and Genomics Laboratory, Peter MacCallum Cancer Centre, East Melbourne, VIC, Australia; ^2^ Sir Peter MacCallum Department of Oncology, The University of Melbourne, Parkville, VIC, Australia; ^3^ Department of Medicine, Royal Melbourne Hospital, The University of Melbourne, Parkville, VIC, Australia; ^4^ Molecular Pathology Research and Development Laboratory, Department of Pathology Peter MacCallum Cancer Centre, East Melbourne, VIC, Australia; ^5^ Translational Genomics and Epigenomics Laboratory, Ludwig Institute for Cancer Research, Olivia Newton-John Cancer and Wellness Centre, Heidelberg, VIC, Australia; ^6^ Tumour Suppression Laboratory, Peter MacCallum Cancer Centre, East Melbourne, VIC, Australia; ^7^ Department of Pathology, University of Melbourne, Parkville, VIC, Australia; ^9^ Department of Gastroenterology, Royal Melbourne Hospital, Parkville, VIC, Australia; ^8^ Department of Biochemistry and Molecular Biology, Monash University, Clayton, VIC, Australia

**Keywords:** Intestinal metaplasia, gastric cancer, TP53, mutation, Mdmx

## Abstract

Intestinal metaplasia (IM) is a premalignant lesion associated with gastric cancer (GC) but is poorly described in terms of molecular changes. Here, we explored the role of *TP53*, a commonly mutated gene in GC, to determine if p53 protein expression and/or the presence of somatic mutations in *TP53* can be used as a predictive marker for patients at risk of progressing to GC from IM. Immunohistochemistry and high resolution melting were used to determine p53 protein expression and *TP53* mutation status respectively in normal gastric mucosa, IM without concurrent GC (IM-GC), IM with concurrent GC (IM+GC) and GC. This comparative study revealed an incremental increase in p53 expression levels with progression of disease from normal mucosa, via an IM intermediate to GC. *TP53* mutations however, were not detected in IM but occurred frequently in GC. Further, we identified increased protein expression of Mdm2/x, both powerful regulators of p53, in 100% of the IM+GC cohort with these samples also exhibiting high levels of wild-type p53 protein. Our data suggests that *TP53* mutations occur late in gastric carcinogenesis contributing to the final transition to cancer. We also demonstrated involvement of Mdmx in GC.

## INTRODUCTION

Gastric cancer (GC) is the fourth most common cancer in the world, with an annual diagnosis of 1 million patients and a 700,000 annual mortality rate [1, 2]. The high mortality rate that is seen globally is mainly due to the advanced stage at diagnosis with few biomarkers for early detection and no systematic screening in the majority of the world outside of Japan and Korea.

**Figure 1:**
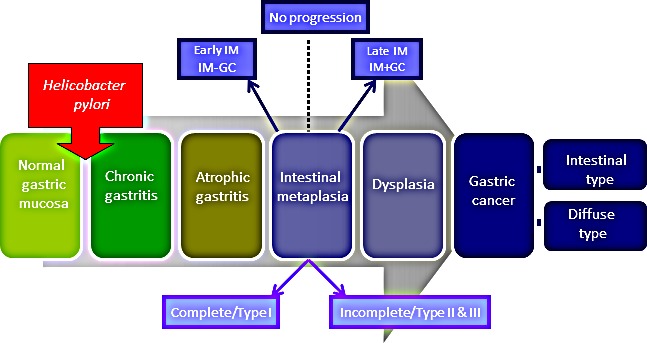
Progressive pathway to Gastric Cancer First proposed by Correa, this schematic diagram outlines the progressive pathway to gastric cancer characterised by distinct histological changes. Normal gastric mucosa is infected with *Helicobacter pylori* which initiates an inflammatory response resulting in chronic, and then atrophic, gastritis. This is followed by intestinal metaplasia (IM) which can be further classified into complete and incomplete subtypes. At this point some patients will then proceed to GC via the intermediate stage of dysplasia. Whilst this model typically describes the pathway to intestinal type GC, these histological changes are often also seen in conjunction with the diffuse subtype of GC. For the purpose of this study IM samples collected adjacent to regions of GC during surgical resection are termed IM+GC- or late stage IM. Similarly IM samples collected from patients with no evidence of GC are referred to as IM-GC or early stage IM.

Whilst the mechanism of gastric carcinogenesis is unknown, a model describing the histological progression of GC was proposed by Correa (Figure 1) [3]. This model predicts that infection with *Helicobacter pylori* triggers [4, 5] a multi-step cascade, with intestinal metaplasia (IM) as a key precursor lesion of GC usually resulting in intestinal type GC (IGC). IM is easily identified histologically by the presence of goblet cells and can be further subdivided, into Types I (complete), II and III (both termed incomplete) depending on the morphology of the cells as well as the types of mucin(s) they secrete. [6]. It has been suggested that incomplete IM (particularly Type III) is more associated with the development of GC [7], however this hypothesis has been disputed by others [8]. Whilst diffuse GC (DGC), the less differentiated subtype of GC, is not often associated with the distinct histological changes described by Correa there does appear to be an association with *H. pylori* and IM (Figure 1) [4].

Whilst the Correa model describes the distinct histological transition from IM to GC relatively little is known about the key genetic events which drive the IM to GC transition. A number of molecular events have been implicated in gastric carcinogenesis (reviewed in [9]). Of particular interest is the involvement of the tumour suppressor *TP53*, the most commonly mutated gene in human cancer [10]. It has been suggested that the frequency of *TP53* mutations increases with the progression of GC from normal gastric mucosa, however, the reported frequency of mutations varies widely between studies [11-13]. This in part reflects the methodology used to define *TP53* mutations with many studies incorrectly using p53 expression as a surrogate for mutations [14]. *TP53* mutations have been shown to be a late event in the progression of some cancer types including colorectal [15]; however this remains to be systematically explored in GC.

In addition to direct mutations of the *TP53* gene, a loss of p53 function can occur through elevation in the levels of one or more of its negative regulators. Among these, Mdmx (also known as Mdm4) and Mdm2 (known collectively as Mdm proteins) are the most powerful regulators of *TP53*. They are frequently altered in human cancer, and their expression is often positively correlated with that of wild-type p53 (reviewed in [16]). Mutations in *TP53* usually result in the accumulation of mutant p53 protein, which disrupts the auto-regulatory loops with the Mdm proteins.

The protection of p53 from these two inhibitors has been shown to be an efficient approach for restoration of tumour suppression by p53 in relevant cancers with elevated Mdm proteins (reviewed in [16]). Recent studies have suggested that elevated levels of Mdm2 are detected in GC compared to adjacent normal tissue and correlate with poor prognosis [17], whilst the involvement of Mdmx has not been investigated to date. To our knowledge, the relationships of Mdm proteins have not been studied in premalignant stages of GC.

The aim of this study was to determine whether p53 protein expression and/or *TP53* mutation status could be used as a biomarker to allow targeted screening of high risk groups with intestinal metaplasia of the stomach. We compared p53 levels and *TP53* mutation status between a cohort of patients with early stage IM (defined as presence of gastric intestinal metaplasia for at least 5 years with no evidence of concurrent GC (IM-GC), and a cohort of IM patients which harboured concurrent GC which we term late stage IM (IM+GC) (Figure 1). This is also the first study to comprehensively analyse the relationship between *TP53* mutation status and its negative regulators Mdm2/x in GC and related precursor lesions.

## RESULTS

### Subtype classification of IM samples

**Figure 2:**
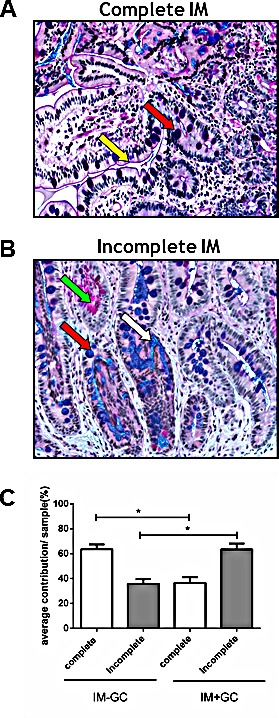
Classification of IM samples All IM samples were stained with Alcian Blue periodic Acid Schiff (ABPAS) to ascertain the predominant IM subtype. The complete type of IM (A) is characterised by goblet cells (red arrow) and a brush border (yellow arrow) whilst the incomplete type (B) is characterised by the presence of neutral (green arrow) and acidic (white arrow) mucins as well as goblet cells (red arrow). (Magnification x200) (C) The percentage contribution of each IM subtype was determined for each IM sample in the cohort. These results were averaged giving the percent contribution of each IM subtype for both the IM-GC and IM+GC groups. Early IM (IM-GC) is more strongly associated with complete IM (p<0.0001; Student's t-test) whilst late stage IM (IM+GC) is more correlated with the incomplete type (p<0.0001; Students t-test). Error bars represent SEM.

In order to determine whether incomplete IM is more associated with the development of GC, we used our study cohort to perform a histomorphological analysis. Complete IM, which most closely resembles the small intestine, is characterised by the presence of a brush border and well defined goblet cells which secrete sialomucins and, in some cases, sulfomucins (Figure 2A). Conversely, incomplete IM more closely resembles the large intestine, lacks a brush border, and comprises sialomucin secreting goblet cells and columnar cells which secrete a mixture of neutral and acid mucins (Figure 2B). All IM samples in our study cohort were stained with AB/PAS and H&E and their respective subtypes were identified based on morphology [18].

We observed that many IM samples in our cohort contain regions of both complete and incomplete subtypes. For each case, the proportion of complete and incomplete IM glands present in each sample was determined and is represented in Figure 2C (see also Table S1). This data suggests that early stage IM samples (see Figure 1), derived from cancer free patients (IM-GC), were comprised predominantly of the complete type (p<0.0001; Student's t-test) whilst late stage IM, from patients with concurrent GC (IM+GC), (see Figure 1) was more closely associated with the incomplete type of IM (p<0.0001; Students t-test).

### p53 immunostaining in normal, premalignant and GC tissue

To assess the expression of p53 within the cohort, sections were stained with an anti-p53 monoclonal antibody (D0-7).

Firstly, a direct comparison of p53 staining was made for the IM components of the two cohorts (Table 1) which were collected from patients without evidence of cancer (IM-GC) or patients with concurrent GC (IM+GC). Of the 62 samples in the IM-GC cohort, 32 (52%) showed positive staining as opposed to the IM+GC cohort where 24/32 samples (75%) showed positive staining (p=0.0451; Fisher's exact test). In both groups, most of the samples only expressed p53 at low levels (82% IM-GC; 78% IM+GC).

**Table 1:** p53 IHC expression in gastric tissue

Comparisons	n=		n=	Negative	Low	High	Positive	p-value negative vs positive	p-value low vs high
**Early and late IM**	**94**	IM-GC	62	30 (48%)	51 (82%)	11 (18%)	32 (52%)	0.0451*	0.7827*
		95% CI		36.4-60.5%	71-89.8%	10.2-29%	39.4-63.6%		
		IM+GC	32	8 (25%)	25 (78%)	7 (22%)	24 (75%)		
		95% CI		13.3-42.1%	61.3-89%	11-38.8%	57.9-86.8%		
									
**IM subtypes**	**86^**	Complete	49	24 (49%)	40 (82%)	9 (18%)	25 (51%)	0.0811*	1*
		95% CI		35.6-62.5%	68.6-90%	10-31.4%	37.5-64.4%		
		Incomplete	37	11 (30%)	30 (81%)	7 (19%)	26 (70%)		
		95% CI		17.5-45.8%	65.8-90.5%	9.5-34.2%	54.2-82.5%		
									
**GC subtypes**	**28^**	Intestinal GC	17	4 (24%)	8 (47%)	9 (53%)	13 (76%)	1*	1*
		95% CI		9.6-47.3%	26.2-69%	31-73.8%	52.7-90.4%		
		Diffuse GC	11	2 (18%)	6 (55%)	5 (45%)	9 (82%)		
		95% CI		5.1-47.7%	28-78.7%	21.2-72%	52.3-94.9%		
									
**Sample type**	**82#**	Normal	82	53 (65%)	77 (94%)	5 (6%)	29 (35%)	<0.0001**	<.0001**
		95% CI		53.8-74.1%	86.5-97.4%	2.6-13.5%	25.9-46.1%	19.26,2	30.49,2
	**94**	IM	94	38 (40%)	76 (81%)	18 (19%)	56 (60%)		
		95% CI		31.1-50.5%	71.8-87.5%	12.5-28.2%	49.5-68.9%		
	**31**	GC	31	7 (23%)	15 (48%)	16 (52%)	24 (77%)		
		95% CI		11.4-39.8%	32-65.2%	34.8-68%	60.2-88.6%		

* Fisher's exact test;

** Chi-square

^ samples of mixed IM subtype were excluded from this analysis (n=8)

! samples of mixed GC subtype (n=3) and those without FFPE blocks (n=1) were excluded

# normal regions are those adjacent to IM samples. In 12 cases the entire tissue section consisted of IM glands and there was no normal tissue available

In order to more thoroughly characterise p53 expression levels in the two histological subtypes of IM, all relevant samples were pooled, irrespective of their cohort of origin, and reclassified based on their predominant histological subtype (Table 1; Table S1). Whilst 51% of the predominantly complete IM samples exhibited positive p53 staining this was increased to 70% in the predominantly incomplete IM samples although this did not reach statistical significance (p=0.0811; Fisher's exact test). Mixed samples, which contained equivalent amounts of complete and incomplete glands, were excluded from this analysis (n=8).

Analysis of this association with respect to intestinal and diffuse subtypes of GC (Table 1) showed that both subtypes of GC exhibit high levels of p53 protein expression with no subtype predilection (p=NS, Fisher's exact test). For one sample the GC FFPE block had been exhausted and this was not analysed. In addition three GC samples exhibited a combination of IGC and DGC subtypes (mixed) and were not included in this analysis.

The overall expression of p53 in benign (normal regions of gastric mucosa, if present in the sample and adjacent to IM; n=82), premalignant (n=94) and GC (n=31) samples within the cohort was assessed (Table 1). This data shows that p53 expression levels incrementally increase with advancing stages of GC progression (p<0.0001; Chi square) and suggests that increased p53 protein expression in IM could be a predictive marker of developing GC.

### *TP53* mutations are only detected in established GC

In order to determine whether increased p53 expression was associated with somatic mutations in the *TP53* gene, HRM analysis for regions covering the entire *TP53* gene was performed. Samples were selected based on the availability of fresh frozen tissue for extraction (see Table S2 for clinical characteristics). For all samples needle macrodissection was performed after pathological review to enrich for areas of IM or cancer. As shown in Table 2, 13 of the 23 GC cases (56.5%) tested were positive by HRM. The identified mutations were then validated and characterised by Sanger sequencing. Of the 13 positive samples, four (30.1%) harboured mutations in exon 7, three (23.1%) in exon 6, three (23.1%) in exon 8, two (15.4%) in exon 5 and one (7.7%) in exon 4. The majority of the detected mutations (7/13 (53.8%)) were missense mutations and predicted to cause a non-functional protein. One patient (P#1816) harboured independent *TP53* mutations in exons 2 and 5. Of the samples analysed, *TP53* mutations were more commonly observed in GC samples of the intestinal subtype (10/15; 66%) compared to the diffuse subtype (2/7; 28.5%).

**Table 2:** Summary of p53 protein expression and *TP53* mutation status in IM+GC and GC samples

					Normal cells		IM		GC			
	Patient#	IM type	Tumour type	T-stage	IHC	HRM	IHC	HRM	IHC	HRM	Mutation location	change
**1**	666	Mixed	IGC	3	0	N/A	0	−	0	+	Ex7	c.723C>-, g.13360C>-,p.?, frameshift
**2**	4891	Incomplete	IGC	3	1	N/A	1	−	3	+	Ex7	c.711G>A, g.13348G>A, p.M2371, missense
**3**	1116	Complete	DGC	3	0	N/A	1	−	3	−	N/A	
**4**	1054	Incomplete	IGC	3	1	N/A	2	−	2	−	N/A	
**5**	9445	Incomplete	IGC	3	1	N/A	1	−	3	−	N/A	
**6**	514	Incomplete	IGC	2	1	N/A	0	−	3	+	Ex8	[c.817 C>T, g.13797C>T, p.R273C + on other allele c.818G>A, g.13798G>A, p.R273H] **OR** [c.817_817delinsTA, g.13797_13798CG>TA, p.R273Y]
**7**	628	Incomplete	IGC	1	0	N/A	0	−	3	+	Ex6	c.659A>G, g.12728A>G, p.Y220C, missense
**8**	597	Incomplete	DGC	1	0	N/A	1	−	3	+	Ex8	c.853G>A, g.13833G>A, p.E285K, missense
**9**	1559	Incomplete	IGC	3	0	N/A	0	−	0	+	Ex6	c.586C>T, g.12655C>T, p.R196X, nonsense
**10**	4715	Complete	IGC	1	0	N/A	1	−	2	−	N/A	
**11**	50	Incomplete	DGC	3	0	N/A	0	−	2	+	Ex6	c.623A>T, g.12692A>T, p.D208V, missense
**12**	51	Incomplete	DGC	3	0	N/A	0	−	1	−	N/A	
**13**	76	Incomplete	DGC	3	0	N/A	0	−	1	−	N/A	
**14**	503	Incomplete	IGC	2	1	N/A	1	−	2	+	Ex8	c.844C>T, g.13824C>T, p.R282W, missense
**15**	4	Complete	IGC	3	0	N/A	1	−	ND	+	Ex4	c.326T>C,p.F109S, missense
**16**	62	Incomplete	Mixed	3	1	N/A	1	−	3	−	N/A	
**17**	450	Incomplete	Mixed	3	0	N/A	1	−	0	+	Ex7	c.782+1G>A, p.?, intron splice
**18**	513	Incomplete	DGC	3	0	N/A	0	−	1	−	N/A	
**19**	1162	Incomplete	Mixed	3	0	N/A	0	−	2	−	N/A	
**20**	1707	Mixed	DGC	2	0	N/A	1	−	2	−	N/A	
**21**	1816	Complete	IGC	2	0	N/A	2	+	1	+	Ex2	c.-11G>A (Homozygous) **AND**
								−		+	Ex5	c.559+1G>A, p.?, intron splice
**22**	8330	Mixed	IGC	3	0	N/A	1	−	1	+	Ex7	c.782+1G>T, p.?, intron splice
**23**	8483	Incomplete	IGC	1	0	N/A	1	−	3	+	Ex5	c.524G>A, p.R175H, missense

ND: no FFPE block available; N/A not applicable

HRM was then performed on the paired IM samples from the GC cases above to determine whether the corresponding mutations were already established in the premalignant stage (Table 3). In most (22/23) IM+GC cases the mutation identified in the GC samples was absent in matched IM. The exception was P#1816 which contained a *TP53* mutation at exon 5 in both the tumour and matched IM sample. Further analysis of peripheral blood DNA obtained from this patient showed this to be a novel germline variant. The observation that no somatic mutations were detected in *TP53* for the IM component of the IM+GC cases provides strong evidence that *TP53* mutation occurs late in the transition event between IM and prior to the development of GC.

**Table 3:** Summary of p53 protein expression and mutation status in IM-GC samples

			IM	
	Patient#	IM type	IHC	HRM
1	10-038-01	Incomplete	3	-
2	2006-009	Incomplete	1	-
3	09-032-01	Incomplete	2	-
4	10-043-01	Incomplete	2	-
5	09-0025-01	Incomplete	1	-
6	2007-0015	Incomplete	ND	-
7	09-0028-01	Complete	0	-
8	08-020	Complete	0	-
9	08-021	Complete	2	-
10	09-021-02	Complete	2	-
11	09-024-01	Complete	2	-
12	2008-0017	Complete	0	-
13	10-037-01	Complete	2	-
14	09-033-03	Complete	2	-

ND: no FFPE block available

To further validate this finding, 14 early stage IM samples i.e., from non-cancer patients (IM-GC) were also screened using HRM. There were no mutations in *TP53* detected in any of these cases. Thus, although p53 expression may be elevated in IM this is wild type protein and harbours no somatic mutations in early or late IM. Mutations were only found in GC.

### Wild-type *TP53* is associated with Mdm2/x expression

Sequence analysis of *TP53* in the patient samples revealed an absence of mutations in premalignant lesions and a strong selection for mutation in a high proportion of GC cases. Expression of p53 protein however increased incrementally with disease progression even in the premalignant stages. In cancers, where expression of wild- type *TP53* is maintained, it is often associated with the expression or amplification of a p53 negative regulator such as Mdm2 or Mdmx. We therefore examined whether Mdm2 and/or MdmX proteins are overexpressed in IM and/or matched GC samples, and how this correlated with p53 expression using serial tissue sections.

Firstly, IM samples were analysed by identifying the region of IM with the highest level of p53 protein expression. The corresponding region was located on the Mdm2 and Mdmx stained serial slides and the respective staining patterns for these proteins were determined (Table S3; representative images in Fig S1). For the purpose of this analysis a sample was considered to have elevated p53 protein expression if greater than 10% of cancer cells were immunopositive (i.e., IHC score of 2+ or 3+; referred to as H in Table S3). A similar 10% immunopositive cutoff was also used to define elevated Mdmx and/or Mdm2 proteins in the same location. Data for Mdm2 and MdmX were then combined and those having elevated levels of one or both proteins classified as positive (Table S3). This data was analysed in combination with the previously generated *TP53* mutation data using criteria described previously [19] to further classify the samples. Results are detailed in Table S3 and summarized in Table 4.

**Table 4:** Summary of p53 and Mdm alterations at genetic and protein levels in IM+GC amd GC samples

		IM+GC (n=21)	
		WT *TP53* (100%)	Mutant *TP53* (0%)
alterations in p53 protein	alterations in Mdm2/x gene(s) or protein(s)		
+	+	3 (14.3%)	0 (0%)
+	−	0 (0%)	0 (0%)
−	+	9 (42.9%)	0 (0%)
−	−	9 (42.9%)	0 (0%)

		GC (n=23)	
		WT *TP53* (100%)	Mutant *TP53* (0%)
alterations in p53 protein	alterations in Mdm2/x gene(s) or protein(s)		
+	+	6 (26.1%)	2 (8.7%)
+	−	2 (8.7%)	6 (26.1%)
−	+	2 (8.7%)	1 (4.3%)
−	−	0 (0%)	4 (17.4%)

In the IM component of patients with associated GC (IM+GC), the *TP53* gene was exclusively wild-type. Elevation of p53 protein was only observed in conjunction with elevated Mdm protein expression (3/21; 14.3%). In the absence of p53 expression, elevated Mdm2/x protein expression was frequently observed (9/21 samples; 42.9%; Table 4) suggesting that p53 is kept in check as predicted through elevated Mdm2/x.

GC samples were analysed in a similar manner however in addition, quantitative real time PCR was used to determine DNA copy number levels of *MDM2* and *MDMX* in GC cases with wild-type *TP53;* Table S3). For this analysis a sample was also considered to be altered in *MDM2/X* if the copy number status for either (or both) of these genes was more than diploid. Again, data for the *MDM* locus were then combined and those having elevated levels of one or both proteins and/or a copy number increase in one or both genes were classified as positive (Table S3).

During the progression to GC, most samples had elevated p53 protein expression, irrespective of *TP53* mutation status. Elevation of p53 protein expression in conjunction with elevated Mdm protein expression and/or *MDM* copy number alterations were more commonly detected in samples with wild-type *TP53* (26.1%; Table 4; Table S3). The majority of the observed Mdm protein changes were due to a preferential elevation of Mdmx rather than Mdm2 expression (Table S3). In cancers harbouring a wild-type *TP53* gene, amplification of *MDMX* was detected in 4/10 (40%; Table S3) samples and was restricted to those of the diffuse or mixed subtypes. Whilst most of these were low level gains of an extra 1-2 copies, one sample harboured high level (>20 copies) copy number gain in both *MDM2* and *MDMX* genes. These results strongly implicate the involvement of *MDMX* in maintaining the wild-type *TP53* status observed in IM.

## DISCUSSION

Poor outcome commonly associated with GC can, in part, be attributed to the late stage of diagnosis of the disease. IM is a well-known pre-neoplastic lesion associated with GC, which is able to persist for many years before progressing to neoplasia. This long latency period between IM and GC [9] represents an ideal time for intervention, however not all patients with IM will progress and thus it is important to be able to identify those patients at risk of developing the disease. Here we show that the incomplete type of IM is significantly more likely to be associated with the development of GC and this subtype is more commonly seen in later stages of the GC progression pathway. This histological subtype of IM can be thought to be a more genomically susceptible stage of disease, and consistent with other studies, more at risk of progression to cancer [7, 18, 20].

Due to the long latency period associated with GC it is conceivable that identification of the key molecular changes which drive the progression to GC in a subgroup of patients could aid clinicians in assigning risk to individuals with IM. Mutations in the *TP53* gene are associated with many cancer types and often inactivate the tumour suppressor activity of the protein, including loss of transcriptional activation of its negative inhibitor *MDM2*. Reduced Mdm2 levels, together with altered degradation processes increases p53 half-life, which subsequently results in the nuclear accumulation of p53 [21]. Antibodies utilised for IHC studies cannot easily distinguish between wild-type and missense mutant p53 proteins, due to loss of conformation during fixation. Most studies on GC and associated premalignant conditions have utilised p53 expression as a surrogate marker for *TP53* mutation (reviewed in [10]) with only a few sequencing the gene. However, since mutant p53 is not always stable and wild-type p53 is also stabilized under stress conditions; existing studies based on IHC staining alone are insufficient, accounting for much of the discordance between reported studies about p53 in GC.

p53 expression in normal gastric mucosa has been reported in only a few studies and was only detected in a small proportion of cells [22, 23]. This is consistent with our study where only between 0-10% of cells were immunopositive in 94% of samples tested. The disconcordance in published results may, in part, be attributed to the recognition sites of the antibody used but is more likely due to differences in the scoring criteria applied to define those samples with positive staining.

Elevated p53 protein levels have previously been reported in both IM and GC [22, 24]. We sought to determine whether p53 expression and/or mutation could be utilised as a biomarker to predict progression of patients with IM to GC. Our results show a significant increase in p53 protein expression during the transition from normal gastric mucosa to IM and then to GC. The level of p53 expression in IM varies among studies from 0-42% [14, 22, 25-27] and appears to differ depending on the type of IM and whether the IM was collected from cancer free patients or those with concurrent GC. This is consistent with our data where p53 expression was higher in IM with concurrent GC.

Our analysis also revealed a switch in *TP53* mutation status between the IM (0%) to GC (56.5%). The frequency of mutations observed in our GC samples is consistent with existing literature and like other studies we also observed that *TP53* mutations were more common in IGC compared to DGC [13, 28]. Our data strongly implicates wild-type p53 as an important checkpoint in the progression of GC suggesting that *TP53* mutation is a pivotal event in the neoplastic transition from IM to GC.

Only two previous studies [11, 24] have reported the presence of mutations in IM samples however, in both studies microdisection was not used to isolate IM prior to analysis which may have resulted in contamination by adjacent tumour cells.

The absence of *TP53* mutations in IM, despite high protein expression, argued that p53 is present, but ineffective possibly due to suppression of its activity. Key regulators involved in the suppression of p53 activity are Mdm2 and Mdmx. Mdm2 protein is the major E3 ligase of p53, acting as the major regulator of p53 protein stability, maintaining p53 as a labile protein with a short half-life [29]. Mdmx interacts with the transactivation domain of p53 and blocks its transcriptional activity however, Mdmx has also previously been shown to compete with Mdm2 for p53 binding, resulting in the accumulation of wild-type p53 under conditions of stress [30]. In contrast to existing literature [19] we observed negligible levels of Mdm2 staining in all IM and GC samples studied. Intriguingly, when examining IM and its matching tumour we observed substantial increase in Mdmx expression in the majority of IM samples (12/21 samples; 57%; Table S3), which further increased in GC samples with wild-type p53 (8/10; 80%; Table S3). These findings strongly implicate Mdmx in IM and suggest that elevation in Mdmx protein expression may occur in IM, suppressing p53 activity. Elevation in p53 levels due to inflammatory response to *H. pylori* may select for elevation in Mdmx levels. This is likely to reflect further elevation in p53 levels as the cells progress from IM to GC and further oncogenic stresses that drive this transition. In this particular study the *H. pylori* status (determined by histology or serology) of the patient did not correlate with the accumulation of *TP53* mutations (Table S2).

The novel finding of the alteration in Mdmx expression in the IM to GC transition particularly in those tumour samples harbouring a wild-type *TP53* gene opens an opportunity for targeted intervention either in chemoprevention of GC or in GC therapy. Reactivation of p53 has been long considered an attractive therapeutic strategy. The proof of principle of this approach has been successfully demonstrated in mouse models whereby temporal restoration of p53 resulted in tumour regression [31-33]. Specifically, the efficacy of protecting p53 from elevated Mdmx and triggering tumour suppression has been demonstrated in a retinoblastoma model [34] and more recently in melanoma [35]. Several therapeutic approaches for the inhibition of Mdmx are currently under development. These include small molecule and stapled peptides designed to inhibit p53-Mdmx interaction, and compounds intended to suppress Mdmx protein expression (reviewed in[16]). Such treatments are predicted to be suitable for IM patients, which express wild-type p53 with elevated Mdmx, and have a strong likelihood of progressing to GC.

**Figure 3:**
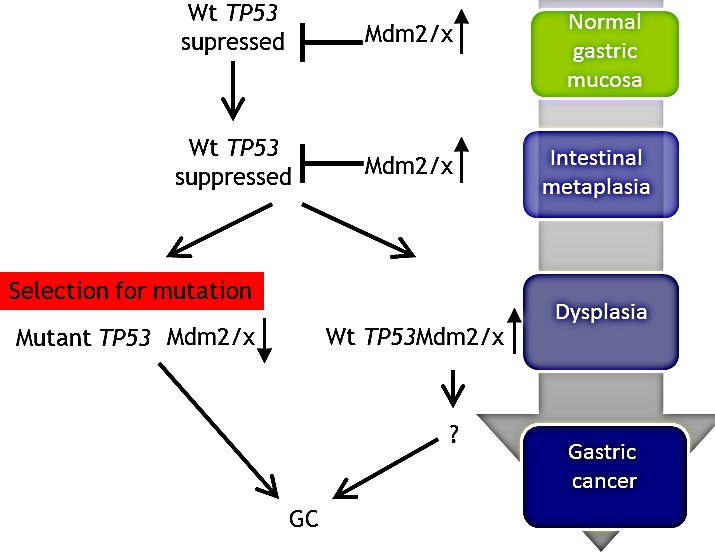
Proposed model for the mechanism of TP53 and MDM2/X gastric cancer In normal gastric mucosa *TP53* is wild-type and its expression is kept in check by Mdm2 and Mdmx. During progression to the intermediate stage of IM the *TP53* gene continues to be maintained in the wild-type state but protein expression levels are slightly elevated, possibly due to a decreasing Mdm2/x expression in a small proportion of cases. Mutations in *TP53* occur at late stage in the progressive pathway in 56% of GC cases, accompanied by a decrease in Mdm2/x levels. In those GC samples which maintain a wild-type *TP53* status Mdm2/x levels continue to remain elevated.

The results described in this study are consistent with the following model involving the Mdmx-p53 axis in disease progression from IM to GC (Figure 3). During IM and in the context of a chronic inflammatory response (possibly due to chronic *H. pylori* infection), p53 is activated and accumulates. This results in a selection for elevation in Mdmx expression in order to suppress senescence by p53. At late stage of progression, most likely during dysplasia, further stress conditions drive a selection for *TP53* mutations, which contributes to the transition to GC. In cases where p53 remains wild-type Mdmx expression remains high and contributes to GC by driving p53-independent pathways.

In conclusion, we describe a model for the frequent overexpression of p53 in premalignant stages of GC and suggest how wild type p53 can be sustained in these non-neoplastic tissues by the expression of the p53 regulator Mdmx. This offers a new target for therapy of GC and a potential avenue for the chemoprevention of GC.

## MATERIALS AND METHODS

### Sample cohort

Gastric IM (IM+GC) and matching tumour samples (GC) were collected from patients undergoing curative or palliative resection for GC from Melbourne, Australia. Tumour sections were obtained from viable tumour tissue. Non-malignant mucosa at least 2cm from the macroscopic tumour margin but from the same anatomical region was also resected and, based on pathological review, those were found to contain regions of IM were selected as the IM+GC cohort. Within 30 minutes of resection, samples were divided for storage into either liquid nitrogen (fresh frozen) or fixed in either formalin or 70% ethanol.

Samples that comprise the IM-GC cohort were gastric biopsies collected during routine endoscopies of patients known to have harboured gastric IM for at least 5 years duration without developing gastric cancer. Biopsies were placed immediately into liquid nitrogen or 10% neutral buffered formalin. Written informed consent was obtained from all patients prior to tissue collection. The procedures carried out in this study were reviewed and accepted by the Institutional Review Board at each of the collection centres.

### IM and GC subtyping

Ethanol or formalin fixed tissue sections were paraffin embedded for histological use. 5 micron sections of each sample were stained with haematoxylin and eosin (H&E) and Alcian-Blue Periodic Acid Schiff (AB/PAS) and histologically reviewed independently by two pathologists. IM samples were assessed for the proportion of complete and incomplete subtypes and GC samples were classified as IGC, DGC or mixed (combination of IGC and DGC) subtypes. These results were compared to the pathology record prepared by the contributing institution. Any variations were further reviewed until a consensus was reached.

### Immunohistochemistry

#### Immunohistochemistry and scoring of p53 stained individual sections

Immunohistochemistry (IHC) for p53 was performed as described in the Supplementary Methods. Nuclear staining of samples were scored as follows: 0 (completely negative), 1+ (<10% cells positive) 2+ (10-49% cells positive) and 3+ (>50% cells positive). For analysis samples were divided into 4 groups: completely negative (score 0), low (score 0 or 1), high (score 2 or 3) and positive (score 1, 2 or 3). For IM samples p53 protein expression was only assessed in the glands with IM. P53 protein expression was quantitated separately for the adjacent non-IM regions and were classified as normal. For GC samples only regions of tissue containing tumour cells were assessed.

#### Immunohistochemistry and scoring of p53/mdmx/mdm2 serial sections

IHC for p53, Mdmx and Mdm2 was performed as described in the Supplementary Methods. In this analysis, assessment of all antigens was made by identifying the region with highest p53 expression and determining the percentage of positive tumour cells. Based on methods described previously by Gunther et al.,[19] a sample was considered to have elevated p53 protein expression if greater than 10% of the cells were immunopositive. The matching region was located in the Mdmx (nuclear staining) and Mdm2 (nuclear membrane staining) stained slides and these were scored in the same manner. A cutoff of 10% positive immunostaining was used to identify elevated Mdm protein expression and a copy number change greater than diploid, determined by RT-qPCR in either *MDM2* or *MDMX* was used to identify genomically altered samples based on methods previously described [19].

### HRM and TP53 sequencing

Fresh frozen tissue was sectioned and macro-dissected to enrich for tumour or IM purity after examination by a pathologist (CM). DNA was extracted using DNeasy tissue kit (Qiagen, Germany). High resolution melting analysis (HRM) was used to screen for mutations in exons 2-11 of the *TP53* gene using the primers previously reported in [36]. Three different normal controls were included in each run and, when available, a positive control sample was included for each amplicon. DNA samples were tested both directly and spiked with normal control DNA (in a sample/control DNA ratio of 3:1). The practice of testing samples admixed with a normal control DNA enables heteroduplex formation, thus allowing more ready identification of homozygous and hemizygous mutations. PCR and HRM were performed on the LightCycler 480 (Roche, Penzberg, Germany). The reaction mixture included 1x PCR buffer containing 1.5 mM of MgCl_2_, 1mM of extra MgCl_2_ (for a final concentration of 2.5 mM MgCl_2_), 200 nM of each primer, 200 μM of dNTPs, 0.25 U of HotStarTaq polymerase (Qiagen, Hilden, Germany), 5 μM of Syto9 (Invitrogen, Carlsbad, CA), either 5 or 2.5 ng DNA (depending on the amount of sample DNA available) and PCR grade water in a total volume of 10 μL. PCR conditions included an activation step of 15 minutes at 95^o^C; 65 PCR cycles (95^o^C for 10 seconds, annealing for 10 seconds comprising 10 cycles of a touchdown from 65 to 55^o^C at 1^o^C/cycle, and extension at 72^o^C for 30 seconds), one cycle of 95^o^C for 1 minute, one cycle of 45^o^C for 1 minute, followed by HRM from 72 to 95^o^C increasing by 0.02^o^C per second. PCR reactions were performed in duplicate. Samples showing a melting profile different to the controls were directly sequenced from a 1/10 dilution of the HRM product using the BigDye Terminator v3.1 cycle sequencing kit (Applied Biosystems, Foster City, CA) according to the manufacturer's instructions. Certain samples, which showed heteroduplexes in multiple amplicons, were re-tested by performing the same PCR/HRM assay after treatment with 0.5 U/reaction of uracil-DNA glycosylase (UDG) to eliminate the templates in which cytosine had been deaminated to uracil, as reported in [37]. The IARC TP53 database was used to identify novel variants.

### Quantitative-PCR (qPCR) for *MDM2* and *MDMX*

*MDM2* and *MDMX* gene copy number was determined as described previously [38]. Briefly, 2ng genomic DNA, 1μM of each forward and reverse primer and SYBR green master mix were used to amplify the gene of interest. Two independent primer pairs were used for each gene. Target gene quantity is given as the average log_2_ ratio value obtained for each primer pair after normalization to the repetitive element Line-1 as a reference gene and a normal DNA reference.

### Statistical analysis

Statistical analysis was performed using Graphpad Prism for Windows (Version 5). Statistical tests used are described in the text and figure legends. In all instances the level of significance was set at p<0.05.

## SUPPLEMENTARY INFORMATION TABLES AND FIGURE


