# Supplementary material for: Role of p53 in the progression of gastric cancer
Source: Oncotarget. 2014 Sep 3;5(23):12016–26. doi: 10.18632/oncotarget.2434 (PMC4322971; doi:10.18632/oncotarget.2434)
Supplement: Supplementary file 1 [file oncotarget-05-12016-s001.pdf]

Role of p53 in the progression of gastric cancer

Supplementary Information

Supplementary Methods:

Immunohistochemistry p53, Mdmx and Mdm2

Immunohistochemical (IHC) staining for p53 was carried out using a monoclonal mouse anti-human p53 antibody (1:200; NCL-p53-DO-7; Novocastra) as the primary antibody. Briefly, paraffin embedded samples were dewaxed in xylene and rehydrated in graded ethanol. Antigen retrieval was performed by heating in a water bath for 30 mins to 100^0C in 10mM citrate buffer. Endogenous peroxidise activity was blocked in 3% peroxidise for 10 mins at room temperature. Sections were then pre-incubated with non-immune goat serum (Vectastain ABC kit, Vector Laboratories, Burlingame, CA, USA) for 1 hour at room temperature, and subsequently with primary antibody in a humidified chamber overnight at 4^0C. After being washed with phosphate buffered saline (PBS), samples were then incubated with a biotinylated goat anti-mouse secondary antibody (1:1000; Vectastain ABC kit, Vector Laboratories, Burlingame, CA, USA) for 1 hour at room temperature. Sections were then incubated with an avidin-biotin horseradish peroxidise complex (Vectastain ABC kit, Vector Laboratories, Burlingame, CA, USA) for 30 mins at room temperature. Immune complexes were visualised by incubation with diaminobenzidine followed by counterstaining with haematoxylin. Slides were then dehydrated and mounted using histological mounting medium. For assessing the immunostaining a semi-quantitative method was used 0 (0% positive cells); 1+ (<10% positive cells); 2+ (10-50% positive cells); 3+ (>50% positive cells).

Three serial sections were cut from formalin fixed paraffin embedded (FFPE) blocks for each sample. One was stained for p53 (as described above) and the remaining sections stained for Mdm2 and Mdmx as follows. The 3 micron sections were dewaxed and hydrated through descending ethanol series and taken to water. Antigen retrieval was performed using pH EnVision ™ FLEX Target Retrieval Solution (Dako, Glostrup, Denmark). Endogenous peroxidase activity in the tissue was quenched with EnVision ™ FLEX Peroxidase-Blocking Reagent (Dako) for 5 minutes at room temperature. The primary antibody, Mdm2 (1:200 Rabbit, Polyclonal, Thermo Scientific) or Mdmx (1:1000 diluted in Ventana antibody diluent buffer containing Dako background reducing diluent; Rabbit polyclonal, Bethyl Laboratory) was applied overnight. Dako Envision Flex in conjunction with Dako DAB enabled detection.

Table S1: Determination of predominant IM subtype

IM-GC

sample #	specimen ID#	complete (%)	incomplete (%)	Predominant IM subtype
1	1999A000189	90	10	complete
2	1999A000256	100	0	complete
3	1999A000559	90	10	complete
4	1999A000569	80	20	complete
5	1999A000853	80	20	complete
6	2000A000014	60	40	complete
7	2000A000084	60	40	complete
8	2000A000134	100	0	complete
9	2000A000135	70	30	complete
10	2000A001035	80	20	complete
11	2001A006544	90	10	complete
12	2002A001390	40	60	incomplete
13	2000A001589	90	10	complete
14	2000A001807	70	30	complete
15	2000A002130	80	20	complete
16	2000A002298	90	10	complete
17	2000A002697	70	30	complete
18	2000A002875	60	40	complete
19	2002A001573	80	20	complete
20	2000A003184	30	70	incomplete
21	2000A003637	80	20	complete
22	2000A003643	70	30	complete
23	2000A003701	70	30	complete
24	2000A003732	50	50	mixed
25	2001A007252	50	50	mixed
26	2000A003955	40	60	incomplete
27	2000A004060	70	30	complete
28	2000A004571	80	20	complete
29	2000A005344	60	40	complete
30	2000A005806	100	0	complete

IM+GC

sample #	specimen ID#	complete (%)	incomplete (%)	Predominant IM subtype
1	1999-00004	70	30	complete
2	1999-00053	20	80	incomplete
3	1999-00054	0	100	incomplete
4	1999-00068	40	60	incomplete
5	1999-00116	0	100	incomplete
6	1999-00182	30	70	incomplete
7	1999-00197	20	80	incomplete
8	1999-00198	20	80	incomplete
9	2001-0008	10	90	incomplete
10	2001-00050	10	90	incomplete
11	2001-0114	50	50	mixed
12	2001-00665	10	90	incomplete
13	2001-00669	25	75	incomplete
14	2002-00073	90	10	complete
15	2002-00131	10	90	incomplete
16	2002-00610	70	30	complete
17	2002-00941	50	50	mixed
18	2002-001106	70	30	complete
19	2003-00630	80	20	complete
20	2003-00843	90	10	complete
21	2003-01492	30	70	incomplete
22	2004-01692	20	80	incomplete
23	2004-01920	40	60	incomplete
24	2004-02356	30	70	incomplete
25	2004-01210	30	70	incomplete
26	2006-01497	50	50	mixed
27	2007-00198	40	60	incomplete
28	2007-00871	40	60	incomplete
29	2008-00330	30	70	incomplete
30	2007-01421	20	80	incomplete

31	2002A004412	90	10	complete
32	2000A005849	90	10	complete
33	2000A006101	20	80	incomplete
34	2000A006271	50	50	mixed
35	2001A000108	70	30	complete
36	2002A000881	70	30	complete
37	2001A000853	40	60	incomplete
38	2001A001991	80	20	complete
39	2001A002899	0	100	incomplete
40	2001A006236	50	50	mixed
41	2002A001106	0	100	incomplete
42	2002A001679	70	30	complete
43	2A003622	100	0	complete
44	2A004412	50	50	mixed
45	2000A000114	90	10	complete
46	2002A004399	30	70	incomplete
47	2002A001966	40	60	incomplete
48	2001A002289	90	10	complete
49	10-038-01	20	80	incomplete
50	2006-009	10	90	incomplete
51	09-032-01	40	60	incomplete
52	10-043-01	40	60	incomplete
53	09-0025-01	30	70	incomplete
54	09-0028-01	80	20	complete
55	08-020	80	20	complete
56	08-021	90	10	complete
57	09-020-02	60	40	complete
58	09-021-02	80	20	complete
59	09-024-01	100	0	complete
60	2008-0017	80	20	complete
61	10-037-01	60	40	complete
62	09-033-03	70	30	complete

31	1999-00084	40	60	incomplete
32	2002-00726	40	60	incomplete

Table S2: Clinical Characteristics of samples based on *TP53* mutation status

Parameter	*TP53* Mutation status	
	**negative (n=10)**	**positive (n=13)**
**IM+GC/GC**		
**Age (years)**		
Male	71.1 (54-95)	69.4 (53-79)
Female	77.5 (76-79)	75 (67-77)
**Gender**		
Male	8	10
Female	2	3
**T Stage**		
T1	1	3
T2	1	3
T3	8	7
***H.pylori* status**		
Positive	7	9
Negative	2	3
Unknown	1	1
**Pathology**		
Diffuse	5	2
Intestinal	3	10
Mixed	2	1
**Associated IM type**		
Complete	2	2
Incomplete	7	9
Mixed	1	2
ND	0	0
	**negative (n=14)**	**positive (n=0)**
**IM-GC**		
**Age (years)**		
Male	61.83 (32-86)	
Female	80.5 (79-82)	
**Gender**		
Male	12	
Female	2	
**Associated IM type**		
Complete	8	
Incomplete	5	
Mixed	0	
ND	1	

Table S3: TP53 and MDM genetic and protein alterations in IM and matched GC

	IM+GC					GC						
Patient #	*TP53* mutation status	p53	Mdmx	Mdm2	Combined MDM alterations	Tumour type	*TP53* mutation status	p53	Mdmx	Mdm2	*MDMX/2* copy number	Combined MDM alterations
666	wild-type	L	L	L	-	Intestinal	mut	L	H	L	not tested	+
4891	wild-type	ND	ND	ND	ND	Intestinal	mut	H	L	L	not tested	-
514	wild-type	L	H	L	+	Intestinal	mut	H	L	L	not tested	-
50	wild-type	ND	ND	ND	ND	Diffuse	mut	H	L	L	not tested	-
628	wild-type	L	H	L	+	Intestinal	mut	H	L	L	not tested	-
597	wild-type	L	H	L	+	Diffuse	mut	H	H	L	not tested	+
1559	wild-type	L	H	L	+	Intestinal	mut	L	L	L	not tested	-
503	wild-type	L	H	L	+	Intestinal	mut	H	L	L	not tested	-
4	wild-type	L	L	L	-	Intestinal	mut	H	L	L	not tested	-
450	wild-type	L	L	L	-	Mixed	mut	L	L	L	not tested	-
1816	wild-type	H	H	L	+	Intestinal	mut	L	L	H	not tested	+
8330	wild-type	L	L	L	-	Intestinal	mut	L	L	L	not tested	-
8483	wild-type	L	H	L	+	Intestinal	mut	H	H	L	not tested	+
						Summary	***Alterations***	***8/13 (61.5%)***	***3/13 (23%)***	***1/13 (7.7%)***	**not tested**	***4/13 (30.8%)***
4715	wild-type	H	H	L	+	Intestinal	wild-type	H	H	L	no change	+
51	wild-type	L	L	L	-	Diffuse	wild-type	L	L	L	CN gain MDMX	+
76	wild-type	L	H	H	+	Diffuse	wild-type	H	L	L	CN gain MDMX	+
1116	wild-type	L	H	L	+	Diffuse	wild-type	H	L	L	no change	-
1054	wild-type	L	H	L	+	Intestinal	wild-type	H	H	H	no change	+
9445	wild-type	H	H	L	+	Intestinal	wild-type	H	H	L	no change	+
62	wild-type	L	L	L	-	Mixed	wild-type	H	L	L	CN gain MDMX	+
513	wild-type	L	L	L	-	Diffuse	wild-type	L	L	L	CN gain MDMX and MDM2	+
1162	wild-type	L	L	L	-	Mixed	wild-type	H	L	L	no change	-
1707	wild-type	L	L	L	-	Diffuse	wild-type	H	H	L	no change	+
Summary	***Alterations***	***3/21 (14.3%)***	***12/21 (57%)***	***1/21 (4.8%)***	***13/21 (61.9%)***	***Summary***	***Alterations***	***8/10 (80%)***	***4/10 (40%)***	***1/10 (10%)***	***4/10 (40%)***	***8/10 (80%)***

ND not determined as no IM present in FFPE block

L (low) protein expression in <10% cells

H (high) protein expression in >10% cells

Combined MDM alterations

+high protein expression (Mdmx and/or Mdm2) or copy number amplification (*MDMX* and/or *MDM2*)

-low protein expression (Mdmx and/or Mdm2) or copy number amplification (*MDMX* and/or *MDM2*)

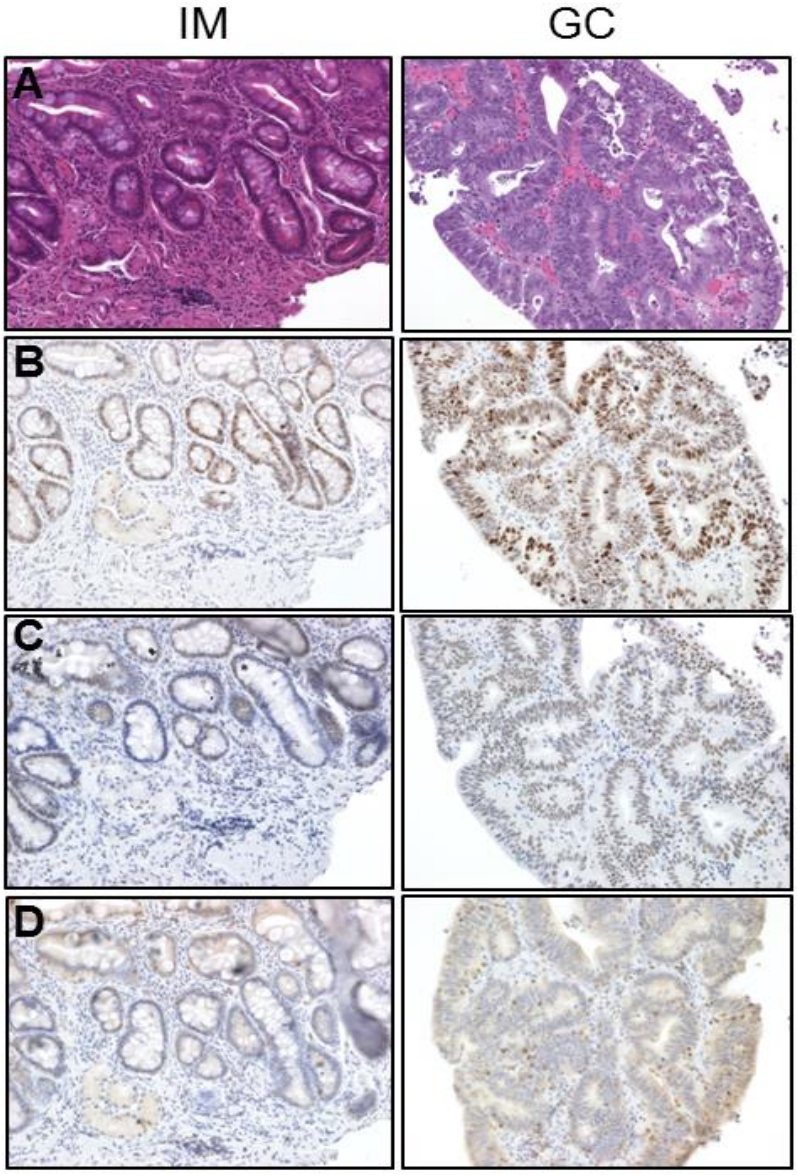

Supplementary Fig 1: Representative images of A) H&E, B) p53, C) Mdmx and D) Mdm2 staining in serial sections from a matched IM and GC pair (Patient #4715)
